# On Lemon Juice in the Treatment of Rheumatism

**Published:** 1851-11

**Authors:** 


*On Lemon Juice in the Treatment of Rheumatism.* By Dr. Golding Bird.—Now, in rhematism, how were we to get rid of this morbid matter in the blood? Opium would remove it to some extent; colchicum would remove it. But it was taking a more common sense view of the subject to employ some remedy which would eliminate the morbific matter from the circulation at once, by carrying it off by the urine. How then, were we to effect this? Why, in acute rheumatism, by the administration of cream of tartar, citrate of potash, lemon-juice, or carbonate of soda. There was little difference between the action of these neutral salts; but lemon-juice, which was the super-citrate of potash, was more quickly absorbed into the blood, and consequently more active in effecting a cure. He had, however, in his own practive, been in the habit of employing the acetate of potash in these cases. This salt, with a mixture of sugar, water, and essence of lemon, acted with marvellous rapidity. In addition to this, given every four hours, he administered five grains of the soap pill, with opium, night and morning; for this not only relieved pain, but prevented the other remedy being carried off by the bowels. These, with the vapor-bath, constituted his treatment of rheumatism, and the result had been always successful. Soda and lemon-juice equally produced an alkaline condition of the blood, but he preferred the acetate of potash, as it was not liable to be neutralized by the presence of acid in the stomach. In respect to the treatment of ague, he never began at once with an anti-periodic. He always disgored the liver by the employment of mild mercurials, and then some quinine. He mentioned one case, however, in which the quinine failed; he then gave the acetate of potash, and the case was cured. By these remedies we immediately altered the character of the blood, and cured the disease — a much more practical and philosophical mode of proceeding than that of treating disease according to name. — *Med. Gaz.*

